# Supplementary material for: An integrative process-based model of fruit growth as a function of carbon and water fluxes modulated by endogenous abscisic acid in blueberry fruit
Source: Quant Plant Biol. 2025 Jun 30;6:e19. doi: 10.1017/qpb.2025.10011 (PMC12277213; doi:10.1017/qpb.2025.10011)
Supplement: Chung et al. supplementary material [file S2632882825100118sup001.docx]

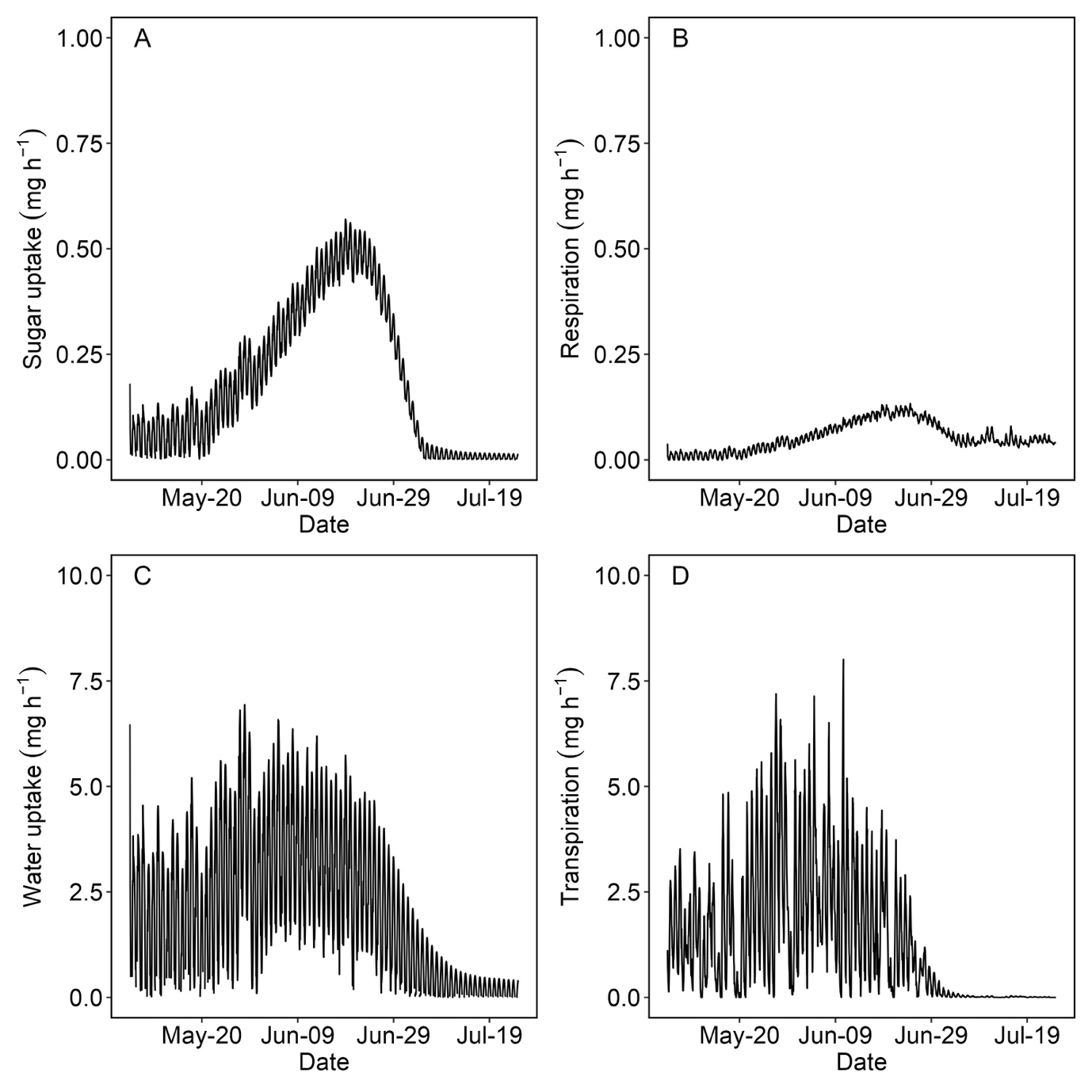


FIG. S1. Simulations of carbon and water fluxes using observation data; sugar uptake (A), respiration (B), water uptake (C), and transpiration (D) of ‘Bluecrop’ blueberry (*Vaccinium corymbosum*) fruit.

**
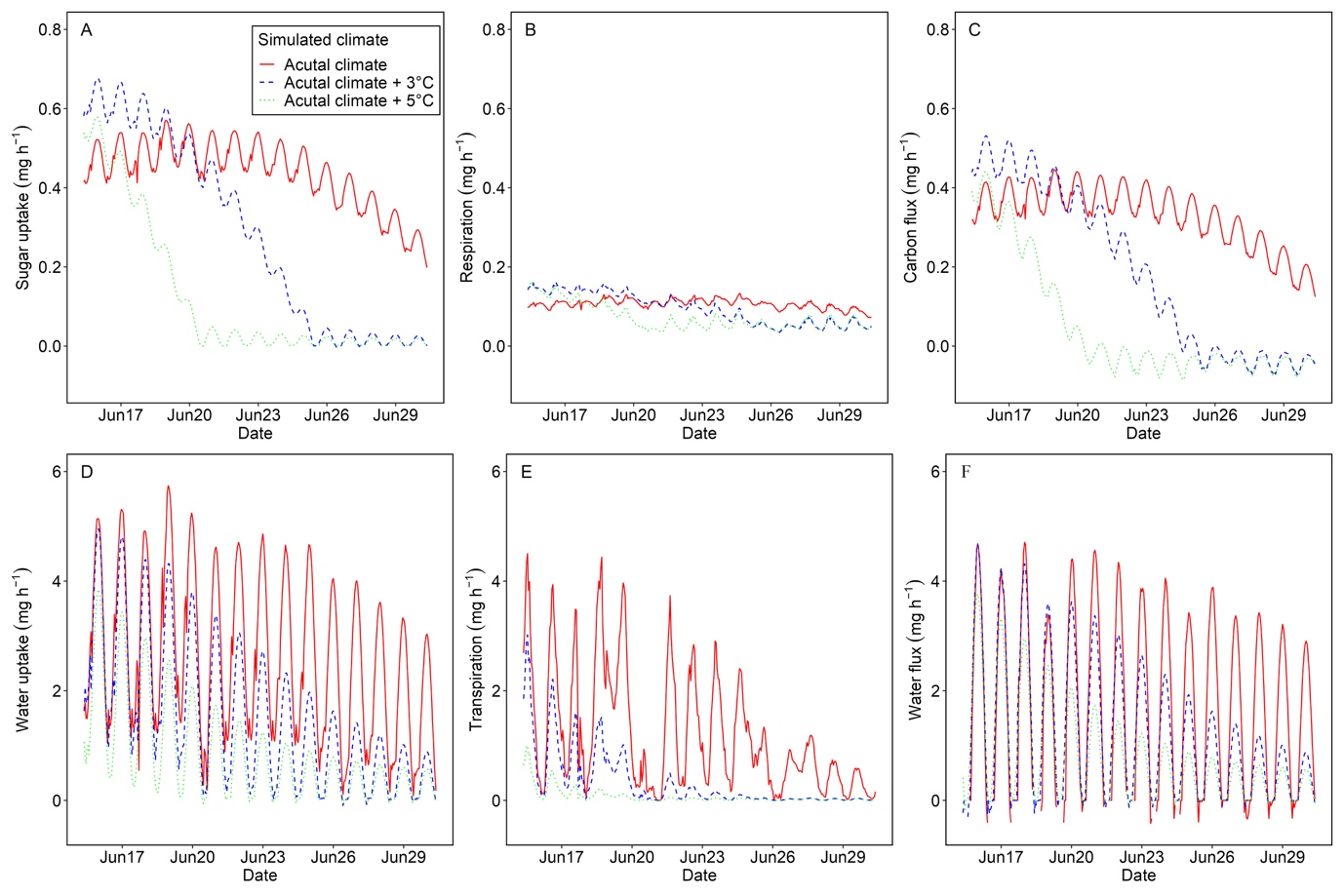
**

FIG. S2. Simulations for the effect of increased average temperature on carbon and water fluxes; the concentration of carbon uptake (A), respiration (B), carbon flux (C), water uptake (D), transpiration (E), and water flux (F) of ‘Bluecrop’ blueberry (*Vaccinium corymbosum*) fruit. These simulations focused on the period between June 15 and June 30, a phase of slowed development as shown in Fig. 4. The actual climate data (red solid lines) represent hourly air temperature and relative temperature from the 2015 season in Suwon, Republic of Korea. For increased temperatures, the hourly temperatures were augmented by +3°C (blue dashed lines) and +5°C (green dotted lines), while relative humidity was maintained consistent across all temperature scenarios.

**
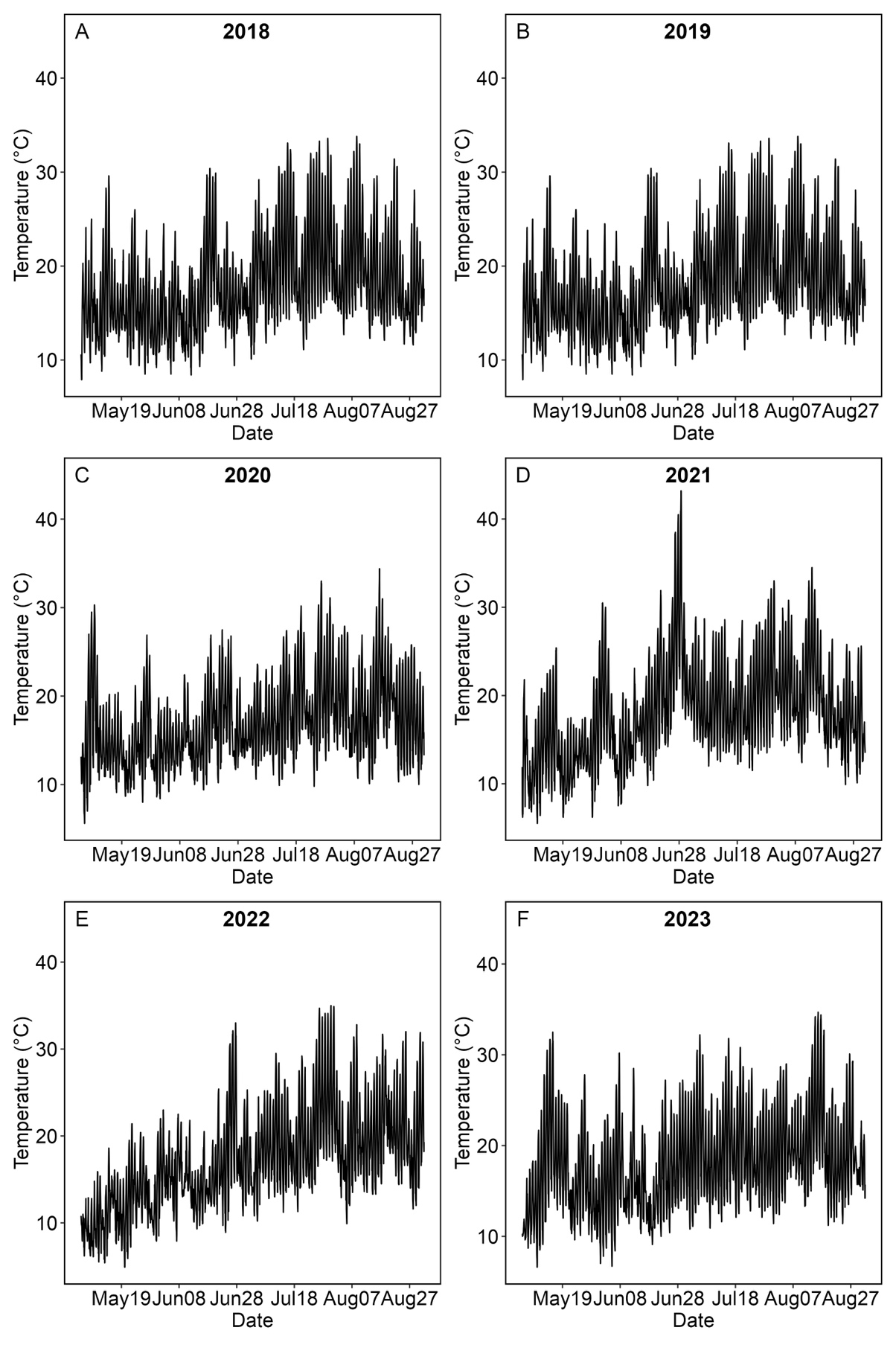
**

FIG. S3. Hourly temperature data from the years 2018 to 2023 in Seattle, USA, sourced from AgWeatherNet (weather.wsu.edu).

**
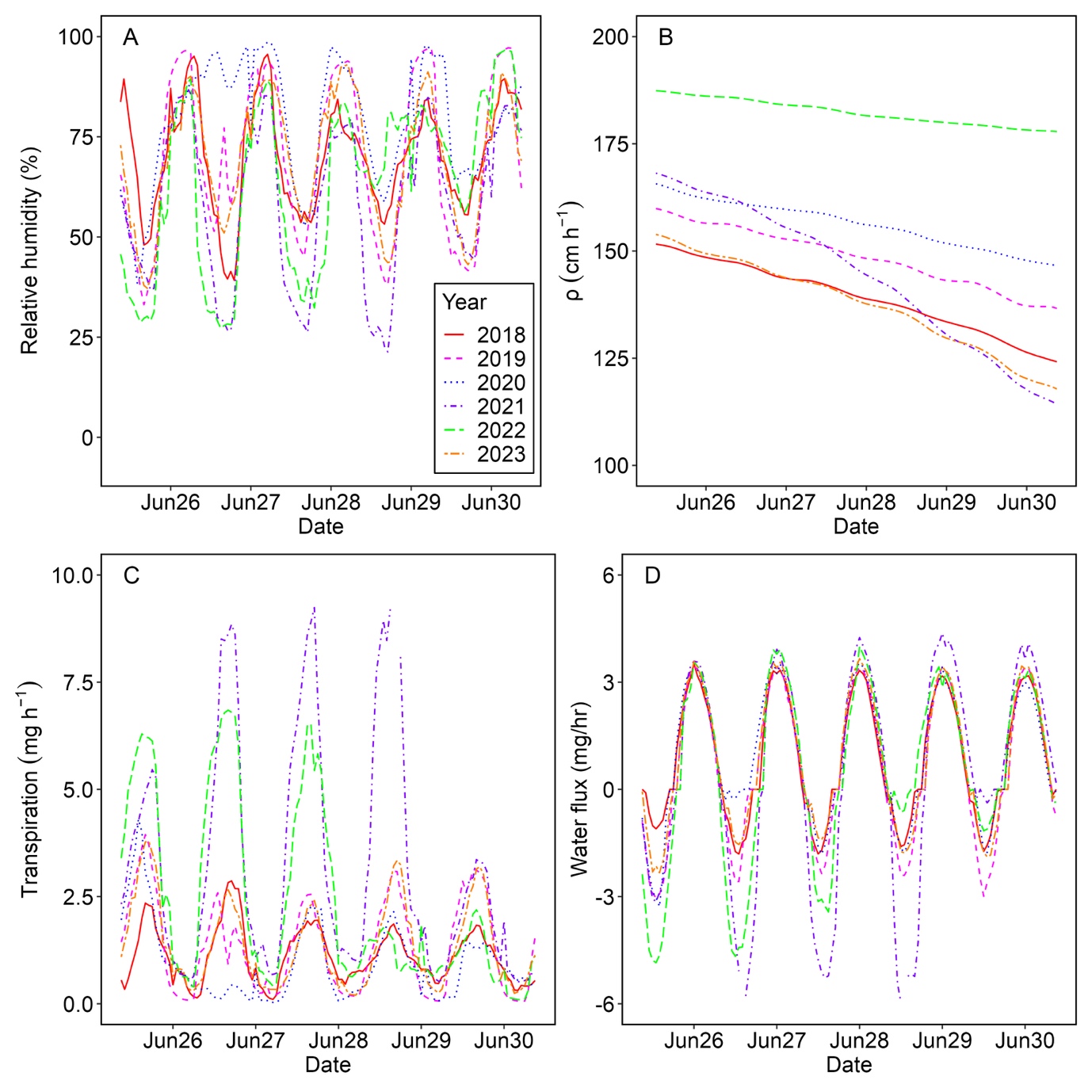
**

FIG. S4. Simulations for the effect of yearly climatic conditions on variables related to water flux; relative humidity (A), *p* is the solute permeability coefficient (B), transpiration (C), and water flux (D) of ‘Bluecrop’ blueberry (*Vaccinium corymbosum*) fruit. These simulations focused on the period between June 25 and June 30; a temporary dip in water mass and fresh mass was observed for the year 2021 and 2023, as shown in Fig. 5. Hourly temperatures and relative humidity were sourced from AgWeatherNet (weather.wsu.edu): 2018, red solid lines; 2019, pink dashed lines; 2020, blue dotted lines; 2021, purple dotted-dashed lines; 2022, green long-dashed lines; 2023, orange two-dashed lines.

**
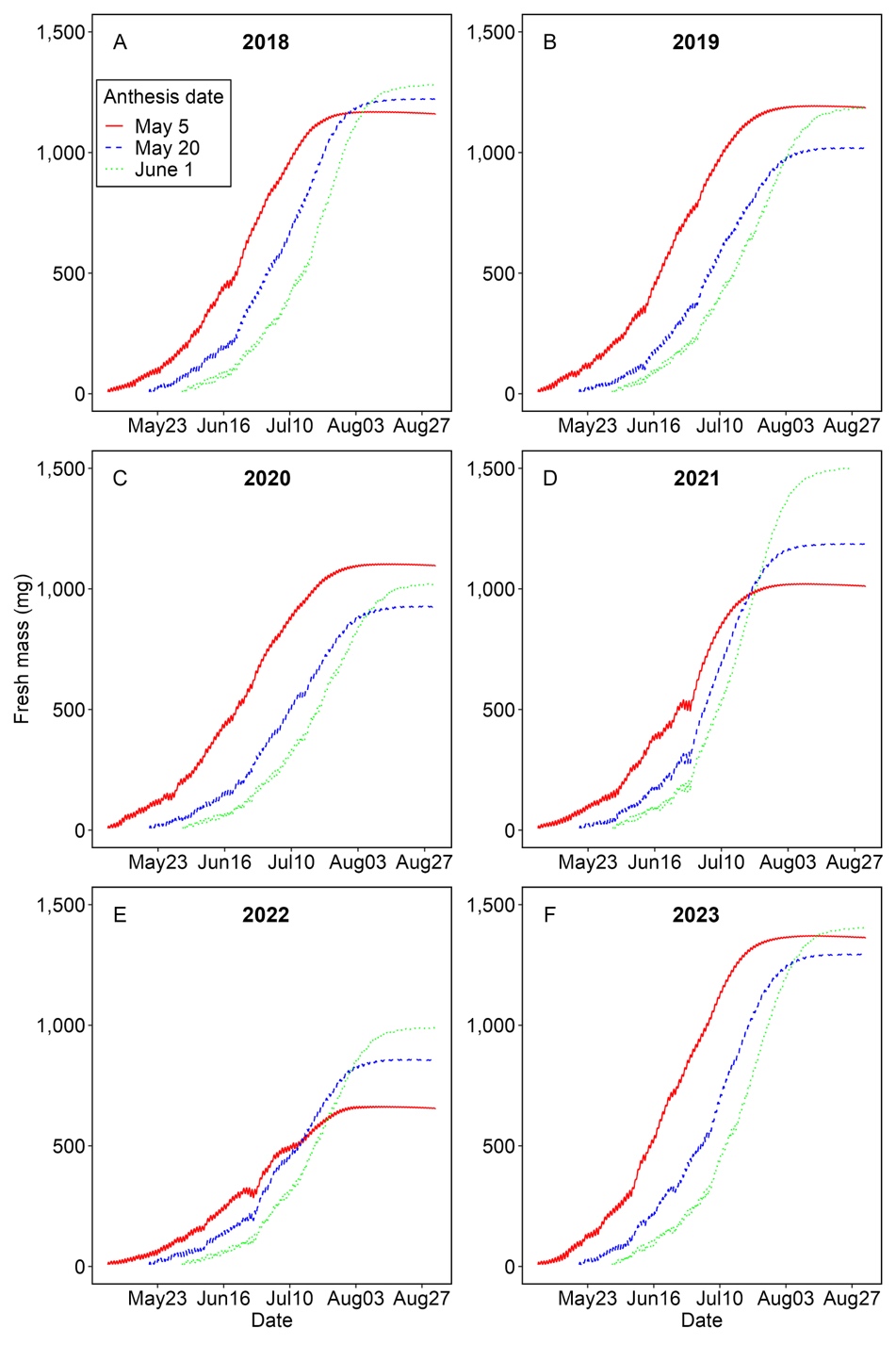
**

FIG. S5. Simulation for the effect of the different timing of anthesis; fresh mass of ‘Bluecrop’ blueberry (*Vaccinium corymbosum*) for the years 2018 to 2023 in Seattle, USA, as retrieved from AgWeatherNet (weather.wsu.edu): May 5, red solid lines, May 20, blue dashed lines; June 1, green dotted lines.

*TABLE S1. List of variables for the model of fruit growth of ‘Bluecrop’ highbush blueberry (Vaccinium corymbosum)*

| Variable | Description |
| --- | --- |
| *A*_f_ (cm^2^) | Fruit surface area |
| *C*_f_ (dimensionless) | Concentration of sugar in fruit pulp |
| *C*_p_ (dimensionless) | Concentration of sugar in phloem |
| *H*_a_ (dimensionless) | Relative humidity in ambient atmosphere |
| *J* (g cm^–2^ h^–1^) | Flow density |
| *P*_f_ (MPa) | Hydrostatic pressure in fruit |
| *P*_p_ (MPa) | Hydrostatic pressure in phloem |
| *P*_x_ (MPa) | Hydrostatic pressure in xylem |
| *R*_f_ (g h^–1^) | Fruit respiration rate |
| *s* (mg) | Dry material in the pulp per fruit |
| *p* (cm h^–1^) | Solute permeability coefficient of fruit skin |
| *T*_f_ (g h^–1^) | Fruit transpiration rate |
| *U*_a_ (g h^–1^) | Active uptake of sugar |
| *U*_p_ (g h^–1^) | Mass flow from phloem to fruit |
| *U*_x_ (g h^–1^) | Mass flow from xylem to fruit |
| *U*_s_ (g h^–1^) | Total rate of sugar uptake |
| *w* (mg) | Amount of water in the pulp per fruit |
| *W*_T_ (mg) | Total fresh mass of fruit |
| π_f_ (MPa) | Osmotic pressure in fruit |
| π_p_ (MPa) | Osmotic pressure in phloem |
| π_x_ (MPa) | Osmotic pressure in xylem |
| Ψ (MPa) | Water potential in stem |
| *ABA_conc_* (μg g^–1^ of *s*) | Concentration of abscisic acid in fruit |
